# Another Myth of Persistence?

**DOI:** 10.1007/s10508-024-03005-1

**Published:** 2024-09-16

**Authors:** Alex Byrne

**Affiliations:** Department of Linguistics and Philosophy, Massachusetts Institute of Technology, 77 Massachusetts Avenue, Cambridge, MA 02139 USA

Gender dysphoria is “the aversion to some or all of those physical characteristics or social roles that connote one’s own biological sex” (Schneider et al., 2009, p. 28). The onset of gender dysphoria may be in early childhood or “around puberty or even much later in life” (American Psychiatric and Association, 2022, p. 517). This Letter concerns childhood-onset gender dysphoria; not gender dysphoria that first manifests in adolescence or adulthood (Zucker et al., 2016). The reported new presentation of “rapid-onset gender dysphoria” (Diaz & Bailey, 2023; Littman, 2018), mostly affecting adolescent natal females, is also not relevant.

In clinical studies, childhood-onset gender dysphoria does not usually persist through puberty, at least if the child has not socially transitioned (Ristori & Steensma, 2016; Singh et al., 2021; Zucker, 2018). If dysphoria persists into puberty, is it unlikely to abate without medical intervention? Many clinicians would give an affirmative answer: “After puberty,” gender dysphoria “seems to be highly persistent” (Gijs & Brewaeys, 2007, p. 213); gender dysphoria “persisting into early puberty appears to be highly persistent” (Cohen‐Kettenis et al., 2008, p. 1895); “in contrast to the low rates of persistence from childhood into adolescence, it appears that the vast majority of transgender adolescents persist in their transgender identity” (Turban et al., 2018, p. 638). In Turban’s (2024) recent book, he writes—in the course of discussing Meyer-Bahlburg (2002)—that “the consensus in the field, even then, was that trans identities were largely stable and unmodifiable after puberty began” (p. 219, fn. †). Gender dysphoria and “transgender identity” are not the same, but a clear implication of these last quotations is that gender dysphoria persisting into puberty will tend to remain unless treated.

The only citation to the first two quotations is Wren (2000); the third quotation, from Turban et al., only cites Cohen-Kettenis and Pfäfflin (2003) (the fourth quotation has no citation). However, Cohen-Kettenis and Pfäfflin (2003) themselves also (and only) cite Wren (2000), quoting this passage:

What does seem to be clear from the research and from clinical descriptions is that, regardless of the numbers who do and who do not successfully obtain surgery, gender-identity disordered adolescents (unlike gender dysphoric prepubertal children) almost invariably become gender-identity disordered adults. (Wren, 2000, p. 222; quoted on p. 172)^1^

To support this claim, Wren gives two citations, Stoller (1992) and Zucker and Bradley (1995). Here is the pertinent passage from Stoller: “Two extensive studies following gender-disordered children for ten to fifteen and more years agree that the majority of gender-disordered children, treated or not, are gender-disordered young adults (Green, 1987; Zuger, 1984)” (p. 86). Note that Stoller says “gender-disordered” adults, not “gender-identity disordered” adults. To see what he means, let’s turn to his two citations.

Zuger followed 55 boys with “effeminate behavior.” When first seen, most were prepubertal and 43 (78%) expressed the desire to be female. The boys were followed for a mean of almost 20 years. As adults, most were clearly gay, and one was a “homosexual transsexual” (Zuger, 1984, p. 93). Zuger does not record whether any others retained cross-sex wishes.

In Green’s (1987) important study, he followed a group of 66 very feminine boys for 15 years. Given his descriptions of this group—“many…had wanted to be girls,” and some “when very young, would say that they *were* girls” (Green, 1987, p. 37)—probably most would have met the current criteria for a diagnosis of “gender dysphoria in children” (American Psychiatric and Association, 2022). Of the 44 Green interviewed as young adults, 32 (73%) were gay or bisexual (Green, 1987, p. 101). Only one was contemplating “sex-change surgery” (p. 115).

Clearly Stoller’s “gender-disordered” adults are not “gender dysphoric” adults but rather same-sex attracted adults (he discusses homosexuality earlier in his paper). Further confirmation is in the continuation of the passage from Stoller quoted above: “One of these projects (Green, 1987) also used controls (non-gender-disordered children); none of the controls has become aberrant gender-wise” (Stoller, 1992, p. 86). Here Stoller’s point concerns sexual orientation: none of Green’s (1987) followed-up controls were homosexual as young adults, although one was bisexual in behavior (pp. 99–100). Wren’s citation to Stoller is thus inapposite.

Wren’s second citation, Zucker and Bradley’s (1995) *Gender Identity Disorder and Psychosexual Problems in Children and Adolescents*, is closer to the mark. However, there is not much support in that book for the hypothesis that untreated dysphoria persisting into puberty is unlikely to resolve. “Clinically,” Zucker and Bradley (1995) cautiously write, “it has been our impression that children who do not move away from extreme cross-gender identification as they enter adolescence may be at greater risk for later transsexualism” (p. 289). But, while “cross-gender identification appears fixed” for some adolescents with gender dysphoria, “for others it may intensify or weaken with time and life events” (p. 302). On the other hand, “Many adolescents continue with chronic gender dysphoria…A few fluctuate between self-definition as homosexual and intermittent wishes for reassignment, typically at stressful times in their lives” (pp. 311–312). This includes adolescents with childhood-onset gender dysphoria: “In our experience, most adolescents with gender identity disorder have had a history of gender identity disorder during childhood” (p. 306).

Zucker and Bradley give some citations reporting that some adolescents can be helped “to accept a homosexual orientation and to relinquish the wish for sex reassignment,” adding the caveat that “the frequency with which such gains have been achieved with severely gender-disordered adolescents is unclear” (p. 315). One of their citations is Lothstein (1980): “Once the major stressors for sex reassignment surgery wishes are identified and labeled, the patients’ urgent demands for surgery often diminish” (p. 103).

To the extent that all roads lead to Wren (2000), there is little evidence that childhood-onset gender dysphoria that continues into early puberty is “highly persistent” if not medically treated. Spack et al. (2012) make the weaker claim that “gender dysphoria in children that *intensifies* with onset of puberty rarely subsides” (p. 419, emphasis added), but this is unsupported by their citation: Zucker (2010). Zucker does mention Wallien and Cohen-Kettenis (2008), which is of some relevance: this paper is discussed below.

Brik et al. (2020) say something similar, albeit more tentatively: “if the gender dysphoric feelings intensify during puberty, they are *thought to be* unlikely to subside” (p. 2611, emphasis added), citing de Vries and Cohen-Kettenis (2012), Hembree et al. (2017), and Zucker et al. (2011).

According to Brik et al.’s first citation, “gender dysphoria rarely changes or desists in adolescents who had been gender dysphoric since childhood and remained so *after puberty* (Cohen-Kettenis & Pfäfflin, 2003; Zucker, 2006)” (de Vries & Cohen-Kettenis, 2012, p. 306; emphasis added). Persisting “after puberty” is obviously not the same as persisting “into puberty.” In any event, Cohen-Kettenis and Pfäfflin (2003) leads to Wren (2000), discussed above. Zucker (2006) leads to Zucker and Bradley (1995), again discussed above, as well as Cohen-Kettenis and van Goozen (1997) and Smith et al. (2001). The latter two are discussed below.

Hembree et al. (2017), Brik’s second citation, states that “the GD/gender incongruence of a minority of prepubertal children appears to persist *in adolescence*” (p. 3876, emphasis added), which is consistent with dysphoria diminishing in adulthood. Hembree et al. cite Steensma et al. (2013b) and Steensma et al. (2011); however, the former cites the latter so Hembree et al.’s two citations are in effect one. Steensma et al. (2011) is discussed below.

Brik et al.’s final citation is Zucker et al. (2011), which states that “there is reasonable evidence that untreated gender dysphoria in adolescents is a relatively stable trait” (p. 59); this is qualified (“reasonable”), and no citation is provided. The comparison is to children with gender dysphoria, so Zucker et al.’s claim is consistent with a substantial proportion of untreated adolescents having reduced or absent dysphoria as adults.

What is needed is a prospective longitudinal study, tracking unmedicalized adolescents with persisting childhood gender dysphoria through puberty into later adulthood. There are no such studies, but one (very imperfect) approximation is Smith et al. (2001), which followed 20 gender dysphoric adolescents who received sex-reassignment surgery and 14 who did not, either because they were refused surgery or didn’t want it. Obviously, the two groups are not comparable; the no-treatment group also had significant comorbidities.

Those without surgery or hormone therapy were assessed at a mean of 17.3 years and 21.6 years at follow-up. Their gender dysphoria had significantly declined from a mean of 46.7 on the Utrecht Gender Dysphoria Scale to 31.1 (range 12–60, with higher scores indicating more dysphoria. Subthreshold for GD corresponds to a mean of around 40: Steensma et al., 2013b). Eleven (79%) in this no-treatment group “did not feel any regrets about having refrained from SR [surgery] or being rejected” (Smith et al., 2001, p. 477). Not much should be made of this result, but it does not favor the “highly persistent” hypothesis. Cohen-Kettenis and van Goozen (1997) is a similar study, but with no follow-up data on the no-treatment group.

Drummond et al. (2008) followed 25 girls who were either diagnosed with gender identity disorder in children according to the DSM-III, III-R, or IV (American Psychiatric Association, 1980, 1987, 1994) or who were subthreshold for such a diagnosis. Mean age at assessment was 8.88 years and 23.24 years at follow-up. Excluding two participants with a disorder of sex development (see Table 3), five were over 12 (up to 12.95) when first assessed and were followed up at ages ranging from 15.44 to 28.72 years. Puberty could have been advanced in these five girls at initial assessment, but the data were not recorded. Two of the five were dysphoric at follow-up: one aged 17.09, the other aged 21.10. The latter was the only one of the five who met all the criteria for a DSM diagnosis. However, another girl who also had a DSM diagnosis was first assessed at 11.26 years; at follow-up, aged 19.27, she was no longer dysphoric.

Wallien and Cohen-Kettenis (2008) tracked 77 children with a mean age of referral to a gender identity clinic of 8.4 years. A total of 58 (75%) had a DSM III-R diagnosis of gender identity disorder (American Psychiatric Association, 1987); the remaining 25% were subthreshold. Fifty-four were followed-up at a mean of 18.9 years. Twenty-one were still dysphoric; the nonresponders (*n* = 23) were presumed to be desisters. All those in the persistence group received an earlier diagnosis of GID; 37 (66%) of the desisters had. Thus, of those with an initial full GID diagnosis, 64% were no longer dysphoric in early adulthood.

Interestingly, the followed-up desisters did not pinpoint early puberty as the crucial period: “In response to our question at what point in time the desisting participants noticed that their cross-gender preferences and feelings had decreased or disappeared, most answered that the change took place upon entry into secondary school. Only few answered that it took place during the first stages of puberty” (Wallien & Cohen-Kettenis, 2008, p. 1422).

Steensma et al. (2011) reported the result of interviews with 25 adolescents (mean age, 15.88 years), all of whom had a DSM-IV or IV-TR diagnosis of GID in childhood (American Psychiatric Association, 1994, 2000). Fourteen had earlier applied for sex reassignment; 11 no longer had gender dysphoria or had it in a mild form. Three factors around ages 10–13 (the end of the children’s elementary school) were identified as important to persistence or desistance: increasing social separation between boys and girls; pubertal changes; and “the experience of falling in love and sexual attraction” (p. 513). The persisters received medical treatment around 12–14; how their dysphoria might have evolved otherwise is unknown.

Steensma et al. (2013a) reported on a prospective longitudinal study of a community sample, with data collected in 1983 (when the participants were children) and 24 years later in 2007. A total of 879 (68%) of the original 1,297 participated in the follow-up, 51 of whom were “gender variant” in childhood (“behaves like opposite sex” and/or “wishes to be of opposite sex”). Fifty reported no “gender discomfort” in adulthood, as assessed by four questions (e.g. “In the past 12 months, did you feel unhappy with the fact of being a man or a woman?”). The single exception was female and “a self-identified lesbian” (p. 2729). Not much to see here: gender variance is a poor proxy for gender dysphoria, and any childhood dysphoria in the 50 might have resolved before puberty (for further discussion of this study, see Abbruzzese et al., 2023, p. 687).

A study of more relevance is Rawee et al. (2024), which examined data from an initial group of 2,772 adolescents over 12 years, from ages 10–12 to 24–26, surveyed at six time points. This was mostly a community sample combined with a smaller clinical cohort (referred for any kind of mental health problem). “Gender non-contentedness” was measured by “I wish to be of the opposite sex.” A total of 2708 responses were recorded at the start, reduced to 1618 (60%) at the end. Using latent class growth analysis (Jung & Wickrama, 2008), Rawee et al. identified a sub-group (19%) as having “decreasing gender non-contentedness,” gradually declining from early puberty to complete gender satisfaction when the respondents were in their early 20s. The details of their model are not particularly important, because prevalence of gender non-contentedness for the entire sample followed the same basic pattern (Rawee et al., 2024, Fig. 1). Gender non-contentedness, like gender variance, is a poor proxy for gender dysphoria. Still, there is no support here for the hypothesis that dysphoria continuing into puberty is unlikely to diminish.

Sapir (2024) used a large national database to investigate the persistence rate of a gender dysphoria diagnosis for minors aged 7.5–17.5 years. Of 34,120 minors with a gender dysphoria diagnosis in 2017 (International Classification of Diseases code F64), 9,144 were billed for at least one health condition of any kind through 2023. Sapir found that fewer and fewer of this latter group were billed for a gender dysphoria diagnosis in the years following 2017, declining to 42.2% in 2023. Finely slicing the group by age would be informative, but Sapir’s data agreement only allowed two coarse age-brackets, 7.5–12.5 years and 12.5–17.5 years. This restriction, as well as the limitations of insurance data mentioned by Sapir, means that the question of this Letter remains unanswered.

For some people with childhood-onset gender dysphoria that persists into puberty, distress will not go away. But for some it will. Money and Russo (1979) followed up five boys “with prepubertal discordance of gender identity/role” in adulthood. All had strong cross-sex wishes during childhood, and all turned out to be gay (perhaps two were bisexual). None had cross-sex wishes at follow-up. One, interviewed at 24, “went so far at the age of 19 as to embark on the real-life test of cross-dressing and presenting himself socially as a woman. After 6 weeks he gave up, having learned from the real-life test that sex reassignment was not for him” (p. 32). It seems that his adolescent dysphoria had petered out in adulthood. Even Green’s (1987) lone “transsexual” was living as a man and lukewarm about a “sex change” when interviewed at 18: “I don’t think about really doing it as much. It’s not as serious. I still want to but I don’t want to go through all the pain or problems and the this and the that. I’m too lazy” (p. 132).

Is childhood-onset gender dysphoria that persists into early puberty—or, alternatively, worsens with early puberty—highly persistent in adolescence and adulthood if untreated? In the opinion of many experts, yes. But the published evidence does not bear this out. The persistence rate, like the detransition rate (Cohn, 2023a), is unknown.

## Data Availability

Not applicable.

## Footnotes

1 Biggs (2023, p. 352) gives the above quotation in this Letter from Cohen‐Kettenis et al. (2008), but then proceeds to discuss what he says is “the only relevant cited source,” which is not Wren (2000), but Smith et al. (2001). Cohen-Kettenis et al. only cite Wren directly after that above quotation; they then continue: “at the Amsterdam gender identity clinic for adolescents, none of the patients who were diagnosed with a GID and considered eligible for SR [surgery] dropped out of the diagnostic or treatment procedures or regretted SR.” At the end of this sentence, Cohen-Kettenis et al. give three other citations: Cohen-Kettenis and van Goozen (1997), Smith et al. (2001), and Smith et al. (2005) (all discussed below). Biggs presumably investigated the citation to Wren and deemed it irrelevant: this Letter makes explicit what Biggs left implicit. See also Cohn (2023b, p. 603), commenting on Rosenthal (2021).
