# Correction: Another Myth of Persistence?

**DOI:** 10.1007/s10508-024-03042-w

**Published:** 2024-11-05

**Authors:** Alex Byrne

**Affiliations:** Department of Linguistics and Philosophy, Massachusetts Institute of Technology, 77 Massachusetts Avenue, Cambridge, MA 02139 USA

**Correction to: Archives of Sexual Behavior** 10.1007/s10508-024-03005-1

In the second full paragraph on the 3rd page of the article, “Forty-four” starting off the 3rd sentence has been corrected to read “Fifty-four”.

In the fourth paragraph on the same page, “Steensma et al. (2011) report” in the first sentence has been corrected to read “Steensma et al. (2011) reported”.

The original article has been corrected.

